# Numerical Simulation of Crack Propagation in Flexible Asphalt Pavements Based on Cohesive Zone Model Developed from Asphalt Mixtures

**DOI:** 10.3390/ma12081278

**Published:** 2019-04-18

**Authors:** Pengfei Liu, Jian Chen, Guoyang Lu, Dawei Wang, Markus Oeser, Sabine Leischner

**Affiliations:** 1 School of Transportation Science and Engineering, Harbin Institute of Technology, Harbin 150090, China; liu@isac.rwth-aachen.de (P.L.); jianchen77@gmail.com (J.C.); 2 Institute of Highway Engineering, RWTH Aachen University, Mies-van-der-Rohe-Street 1, D52074 Aachen, Germany; lu@isac.rwth-aachen.de (G.L.); oeser@isac.rwth-aachen.de (M.O.); 3 Institute for Urban and Pavement Engineering, Technical University of Dresden, Georg-Schumann-Street 7, D01187 Dresden, Germany; Sabine.Leischner@tu-dresden.de

**Keywords:** asphalt pavement, finite element method, cohesive zone method, crack propagation, pavement design

## Abstract

To give engineers involved in planning and designing of asphalt pavements a more accurate prediction of crack initiation and propagation, theory-based models need to be developed to connect the loading conditions and fracture mechanisms present in laboratory tests and under traffic loading. The aim of this study is to develop a technical basis for the simulation of fracture behavior of asphalt pavements. The cohesive zone model (CZM) approach was applied in the commercial FE software ABAQUS to analyze crack propagation in asphalt layers. The CZM developed from the asphalt mixtures in this study can be used to simulate the fracture behavior of pavements and further optimize both the structure and the materials. The investigations demonstrated that the remaining service life of asphalt pavements under cyclic load after the initial onset of macro-cracks can be predicted. The developed CZM can, therefore, usefully supplement conventional design methods by improving the accuracy of the predicted stress states and by increasing the quality, efficiency, and safety of mechanical design methods by using this more realistic modeling approach.

## 1. Introduction

To give engineers involved in planning and design a more accurate prediction of crack initiation and propagation in asphalt pavements, theory-based models need to be developed to connect the loading conditions and fracture mechanisms present in laboratory tests and under traffic loading. Numerical simulations offer excellent possibilities for solving these problems [1,2]. In recent years, various techniques have been developed which implement the laws of fracture mechanics within the framework of the finite element method (FEM) algorithms [3,4,5,6,7,8,9,10]. With the aid of numerical simulations, the propagation of fatigue-induced cracks can be better understood, which in turn improves the predictions of crack formation on the entire pavement structure.

The crack propagations were modeled in structures using the so-called Paris law, which relies on stress intensity factors which account for the large increase in stress at the crack tip [11]. However, the concept of stress intensity factors only applies to ideally elastic materials and cannot be used directly for viscoelastic materials, such as asphalt materials [12]. For this reason, a cohesive zone model (CZM) was developed in [13,14,15,16,17,18], which considers the energy-balance related processes of crack propagation in viscoelastic materials.

The idea of the CZM is based on the assumption that the material separation takes place solely in a narrow strip-shaped zone in front of the crack (cohesive zone) during the fracturing process. According to this concept, the damage of the material essentially takes place in this limited area up to the final separation. The remaining material is free of damage and complies with normal laws of deformation so that it can be modeled without discontinuities, i.e., without separation. Because of its versatility and simple numerical implementation, CZM can be used to simulate crack propagation due to fatigue of viscoelastic or plastic material. Xu and Needleman [19] developed the CZM technique during the 1990s to implement a potential-based CZM by introducing cohesive elements into a finite element mesh with an exponential cohesive law. The successful and effective implementation of CZM into FEM has resulted in significant advances in the field of fracture mechanics of asphalt pavement materials. Soares et al. [20] simulated the indirect tensile test (IDT) for opening mode crack propagation in asphalt mixtures using a CZM. Paulino et al. [21] developed a CZM based on energy potential for hot-mixed asphalt (HMA). This CZM is intrinsic exponential, which has higher computational efficiency and reliability to be applied in FEM. To alleviate the compliance problem introduced by the CZM, Song et al. [22] developed a bilinear CZM by adjusting an initial traction–separation slope for simulation of opening mode and mixed mode fractures in HMA. Baek and Al-Qadi [23,24] used a bilinear CZM to simulate a modified single-edge notched beam (SENB) test with an interface in between of the two HMA layers. Viscoelastic material properties were considered in this simulation. Further development for simulation of the viscoelastic fracture behavior of HMA was carried out by Kim et al. [25]. Wagoner et al. [26] proposed a δ_25_ parameter in the disk-shaped compact tension (DCT) test, which is a type of crack tip opening displacement (CTOD) measured at a close crack tip. This measuring method can minimize the effect of viscoelastic bulk material on the fracture energy measurement. Using the δ_25_ parameter, Song et al. [27] evaluated a power-law CZM for HMA at −20 °C. Wang et al. [28] conducted 2D simulations of unidirectional tension tests with generated aggregate within the bounds of linear elastic and observed the influence of cohesive and adhesive failure on the overall crack resistance. Liu et al. [29] conducted a 2D IDT with aggregate from X-ray CT images to effectively compare the interfacial fracture behaviors of asphalt samples manufactured with different compaction methods. Hu et al. [30] simulated the unidirectional compression test with a 3D geometric reconstruction based on X-ray CT images to analyze the interface stripping damage between the aggregates and asphalt mortar. Yin et al. have conducted numerous 2D simulations with cohesive zone elements (CZE) [31,32,33]. The results showed that the load-displacement curves are affected by the cohesive strength and the fracture energy differently, which are two parameters of CZE. This research group also conducted analogous 3D simulations and found that the 3D simulations result in much higher peak loads corresponding to higher displacements compared with 2D simulations. Souza et al. [34] evaluated the influence of aggregate angularity and binder content on the fracture behavior based on the IDT. The binder content was found to have a positive relationship with the rutting resistance and fatigue performance while the aggregate angularity exhibited an opposite influence.

Although some progress has been made in CZM techniques and its application in the asphalt mixtures, further research is still required to develop the relationship between the fracture behavior in asphalt mixtures and asphalt pavements with CZM. The objective of this research is the development of a basis for the simulation of fracture processes in asphalt pavement structures based on the fracture parameters of the CZM determined from asphalt mixtures. First, the fracture properties of various asphalt mixtures for different asphalt layers in the pavement were determined in laboratory tests and simulations. The computational and experimental crack growths were compared between each other to verify the developed numerical models with regard to its accuracy. Afterwards, the fracture behavior was assessed in a standardized multi-layered asphalt pavement structure by means of FE simulations. The crack propagation in real pavement structures due to traffic loads was used to make evaluate the distribution of cracks and the crack growth throughout the whole cross section.

## 2. Experimental Preparation

### 2.1. Material Design

Crack propagation was investigated on three types of asphalt materials (AC 22 TS, AC 16 BS, and AC 8 DS), which included a base, binder and surface layer. They were prepared in accordance with the German guideline ZTV Asphalt-StB 07/13. Drill cores with a diameter of 150 mm were taken from test plates measuring 300 × 200 × 65 mm^3^ and then ground to a height of 60 mm. The gradations are shown in Table 1.

### 2.2. Constitute Law for Fracture Behavior

**Figure 1:**
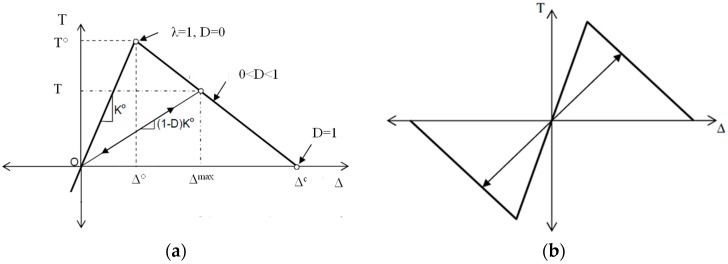
Schematic depiction of typical bilinear traction–separation law. (**a**) normal component, (**b**) tangential component.

The fracture behavior was implemented in ABAQUS and followed a linear softening law. A bilinear traction–separation law defined the constitutive response of the cohesive elements, as shown in Figure 1. 

In the course of crack development, the traction at the interface increases constantly up to a limit value. After that, the traction decreases continuously and finally decays to zero. Thus, a bilinear traction function was selected to describe the traction function T(∆). Three parameters are included in this approach, namely the cohesive fracture energy G, the cohesive strength T°, and the separation ∆ [35]. The cohesive fracture energy G can be expressed as:

(1) $$G=\int_{0}^{\Delta }T\left(\Delta \right)\;d\Delta =\frac{1}{2}T{}^{\circ}\cdot \Delta$$

The onset and development of damage at the interfaces were described with the maximum stress criterion in this study [15,27]. If coupling behavior between different directions is disregarded, the onset of damage will occur when an arbitrary separation value, normalized to its maximum, reaches one, which is expressed as:

(2) $$\lambda =\mathrm{MAX}\left\{\frac{\langle T_{1}\rangle }{T_{1}^{{}^{\circ}}},\;\frac{\langle T_{2}\rangle }{T_{2}^{{}^{\circ}}},\;\frac{\langle T_{3}\rangle }{T_{3}^{{}^{\circ}}}\right\}=1$$

whereby λ is a dimensionless damage initiation parameter, $T_{1}$, $T_{2}$, and $T_{3}$ are stresses in the normal direction and the first and second tangential directions, respectively. The Macaulay bracket operator < > is used to exclude negative (compressive) values since compressive fracturing is not accounted for in damage initiation.

After the damage criterion is reached, the progress of damage is modeled by reducing the stiffness of the cohesive elements. The damage evolution is determined based on the dissipated fracture energy. The fracture energy is equal to the area under the traction–separation curve, as shown in Figure 1. The damage variable, corresponding to the total damage, is the stiffness degradation parameter D which ranges from zero to one and affects the traction and stiffness within the interfaces:

(3) $$T=\left(1-D\right)K^{{}^{\circ}}\Delta$$

(4) $$D=\frac{\Delta^{c}\left(\Delta^{\max}-\Delta^{{}^{\circ}}\right)}{\Delta^{\max}\left(\Delta^{c}-\Delta^{{}^{\circ}}\right)}$$

where $\Delta^{c}$ is the effective separation at complete failure, $\Delta^{\max}$ represents the maximum effective separation during the loading history and $\Delta^{{}^{\circ}}$ is the effective separation at damage initiation.

### 2.3. Static Three-Point Semi-Circular Bend Test

**Figure 2:**
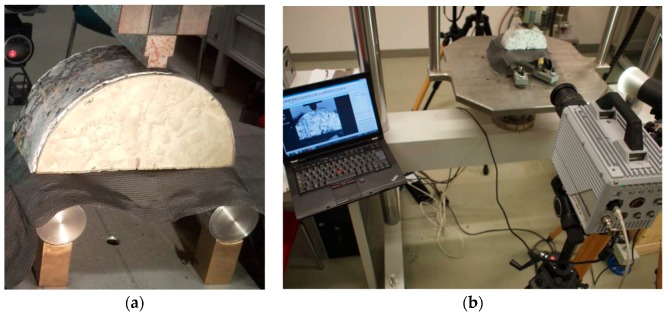
Experimental setup of the static three-point semi-circular bend test (3PSCBT). (**a**) Test setup of the 3PSCBT, (**b**) setup for crack detection.

The initial values for the parameters T° and G were determined by means of a static three-point semi-circular bend test (3PSCBT) with asphalt specimens according to DIN EN 12697-44 [36]. The 3PSCBT uses test specimens with the dimensions of diameter 150 mm and thickness 60 mm. A wider notch (3 mm) with a depth of 9 mm was used in the test. The diameter of the lower supports was 35 mm with a central distance of 120 mm. The upper loading strip had a width of 10 mm and a diameter of 150 mm. The specimens were heated to the temperature of +5 °C for 4 h and then placed in the middle of the supports at +5 °C in the laboratory. The load was applied at a constant displacement rate of 5.0 mm/min. During the testing, the fracture process was recorded by a high-speed camera (FastCam SA 5) every 0.01 s. The applied force and the crack tip opening displacement (CTOD) were used to determine the necessary parameters. The experimental setup of 3PSCBT is shown in Figure 2.

The results of the static 3PSCBT were used to determine the parameters under consideration of the concept of dissipated fracture energy [15,27]. The initial parameters for the CZM were then calibrated until the measured force-displacement curve fits the calculated curve sufficiently well with the aid of the least square method. The final parameters are listed in Table 2. It should be noted at this point that the listed fracture parameters are related to the given test conditions. If the test equipment or the geometry of the test specimens changes, these parameters may require adjusting [35]. Additionally, the parameters for visco-elastic material behavior, such as asphalt, are dependent on temperature and loading speed. Therefore, there is still a need for further investigation of the crack development of the asphalt materials under different temperatures and loading scenarios in order to enable an objective assessment of the fracture behavior.

### 2.4. Dynamic Three-Point Semi-Circular Bend Test

Besides the static 3PSCBT, the dynamic 3PSCBT was also carried out in this study. Its experimental setup corresponds to the static 3PSCBT, as described above.

The specimens were heated to T = +5 °C and then tested around +5 °C in the laboratory. The load was force-controlled with sinusoidal pressure. The frequency was uniformly 10 Hz. The load was given as lower and upper stresses shown in Table 3. The upper stresses were determined as a function of the maximum stresses from the static 3PSCBT.

**Figure 3:**
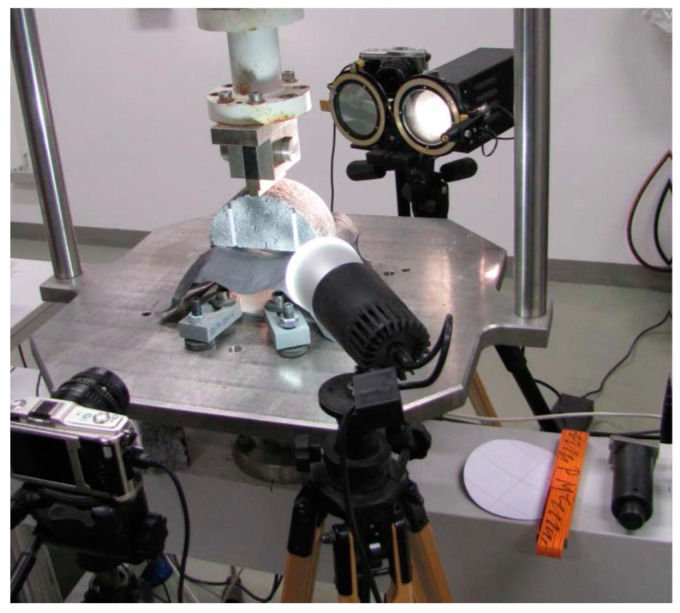
Experimental setup of the dynamic 3PSCBT.

During the tests, the deformation was recorded at a measuring rate of 0.001 s. The data were processed during the measurements for each cycle (calculation of the force and displacement amplitudes) and stored. The test duration was a maximum of 28 min. The experimental setup is shown in Figure 3.

## 3. Numerical Simulation of Crack Propagation in Static 3PSCBT

### 3.1. Construction of the FE Model for Static 3PSCBT

**Figure 4:**
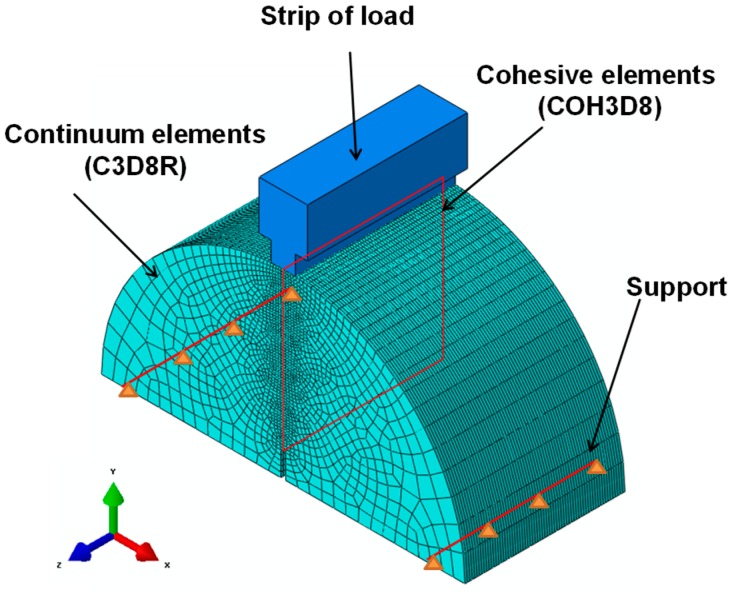
Model for the test specimen of the 3PSCBT.

The three-dimensional (3D) FE model for the test specimen of static 3PSCBT including three different asphalt materials is shown in Figure 4.

The geometry of the model is defined according to the aforementioned description of the 3PSCBT. This model contains 101,112 eight-node continuum elements with reduced integration (C3D8R) and 47,740 eight-node cohesive elements (COH3D8). For the continuum elements, linear-elastic behavior is assumed. The mesh generator in ABAQUS discretizes the cross-section of the specimen into different areas in the horizontal direction. Depending on the distance to the intended crack, the edge length of the elements to the edge areas increases. The cohesive elements have an edge length of 0.3 mm in the y- and z- directions. With a sufficiently fine degree of discretization in the potential crack path, the behavior in the crack region can be represented accurately. The definition of the materials parameters has been introduced previously. The lower supports are simplified as two lines of nodes selected from the specimen in whom the displacement is constrained in the vertical direction. The strip of the load is assumed as a rigid body and the load is defined at a constant displacement rate of 5.0 mm/min according to the aforementioned information about the static 3PSCBT.

### 3.2. Verification of the FE model for static 3PSCBT

**Figure 5:**
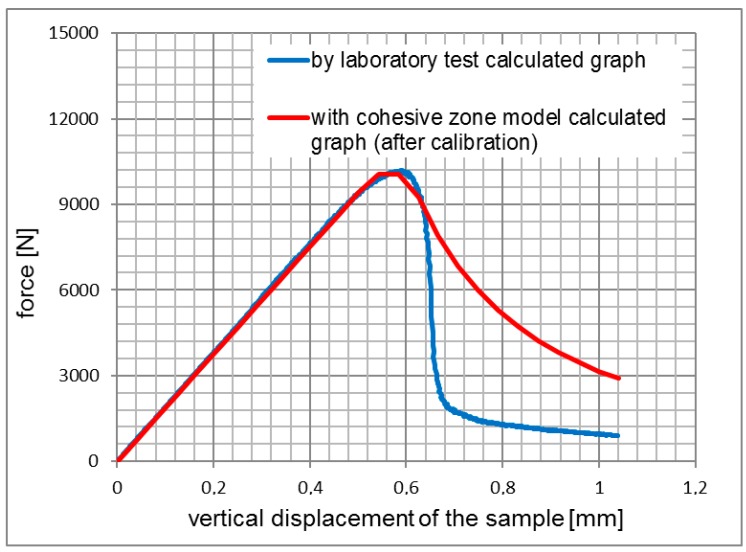
Comparison of the force-displacement curves from asphalt surface layer.

**Figure 6:**
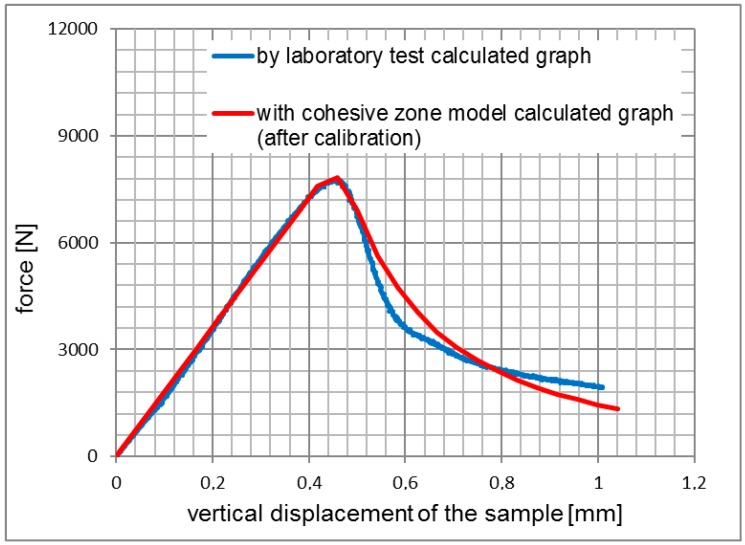
Comparison of the force-displacement curves from asphalt binder layer.

**Figure 7:**
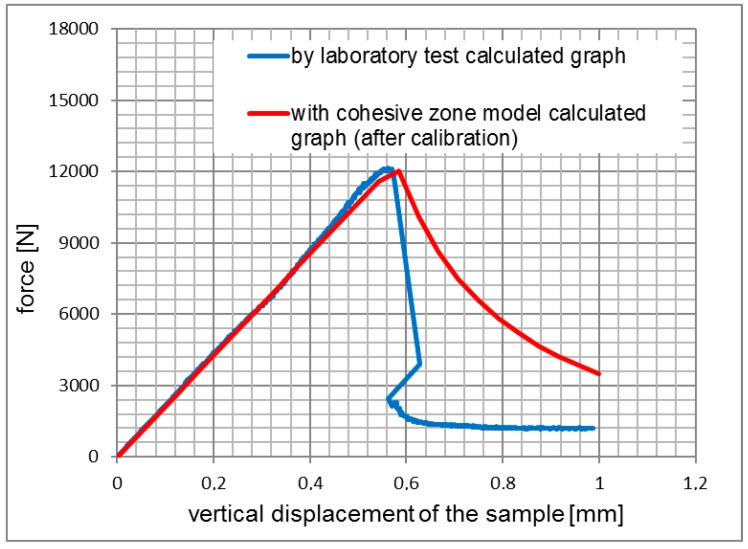
Comparison of the force-displacement curves from asphalt base layer.

The results of the simulations for 3PSCBT were verified by comparing the experimental results, as shown from Figure 5, Figure 6 and Figure 7. Generally speaking, it can be seen that the force-displacement curves derived from the simulation and experiment are consistent with each other, especially for the rising straight part. After the maximum force is reached, where the specimens are failed, the force significantly drops with increased displacement. 

**Figure 8:**
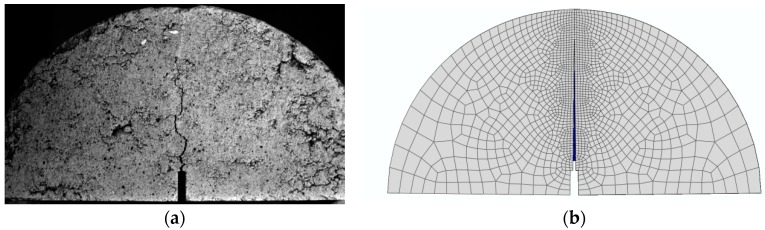
Comparison of cracks between the test and simulation derived from asphalt surface layer at 1 s (**a**) crack in the test, (**b**) crack in the simulation.

The differences between the curves are visible, in particular for the samples of the asphalt surface layer (see Figure 5) and the asphalt base layer (see Figure 7), which means that the cracks in the 3PSCBT propagate a little faster compared to the cracks simulated in the CZM after the failure of the specimens. The CZM in ABAQUS assumed a homogeneous material of the specimens. However, the inhomogeneity of the asphalt materials would cause the deviations between the simulation and the examinations [37,38], as shown in Figure 8. To derive the information of crack initiation and propagation before the failure of the specimens, which is the main purpose of this static 3PSCBT simulation. The computational results are reliable, and therefore, they can be used in the further study.

## 4. Numerical Simulation of Crack Propagation in Pavement Structure

### 4.1. Construction of the FE Model for the Pavement Structure

For the modeling of the pavement, a typical structure for heavy traffic loads was selected according to German rules and regulations (RStO 12). The layer thicknesses and the parameters for the material properties are listed in Table 4. The thickness of the semi-infinitely extended subgrade was defined to be 7000 mm in order to limit the influence of the boundary condition on the results. The length of the entire pavement structure is defined to be 8500 mm in the simulation to limit the required computational time for the mesh generation and calculations. A full layer bond exists only between the asphalt layers. The asphalt base course and the sub-base, as well as the sub-base and the subgrade, are not fully bound and the displacement in horizontal directions is permitted [39].

Since the 3D simulation under cyclic load is extremely time-consuming in ABAQUS, the crack propagation was analyzed with a two-dimensional (2D) CZM under quasi-static loading with a width of 100 mm. The quasi-static loading is displacement driven and is set to progress at 5.0 mm/min, as in the static 3PSCBT.

**Figure 9:**
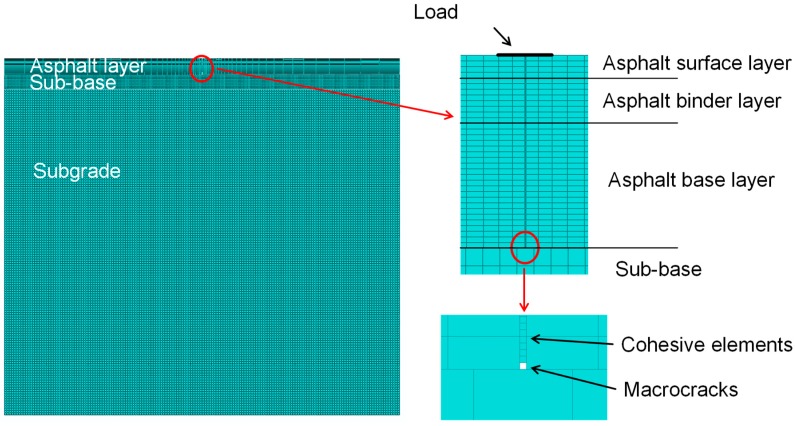
Model of the asphalt pavement.

In the numerical simulation, a macro-crack with a width of 2 mm is assumed at the bottom of the asphalt base layer before the load is initiated. The length of the cohesive elements is set to be 0.3 mm. The details can be found in Figure 9.

### 4.2. Simulation of Crack Propagation in Pavement Structure

**Figure 10:**
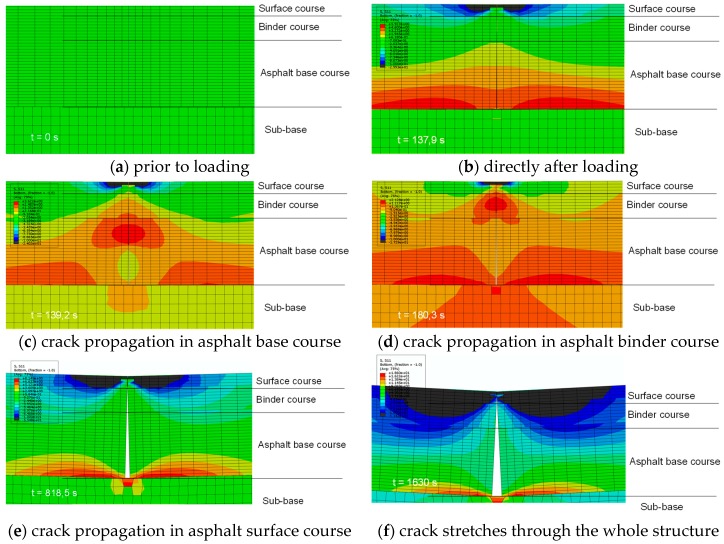
Stress distribution and crack propagation in numerical simulation with cohesive zone elements in ABAQUS (scale factor 100).

Figure 10 shows the stress distribution in the horizontal direction and the crack propagation. 

**Figure 11:**
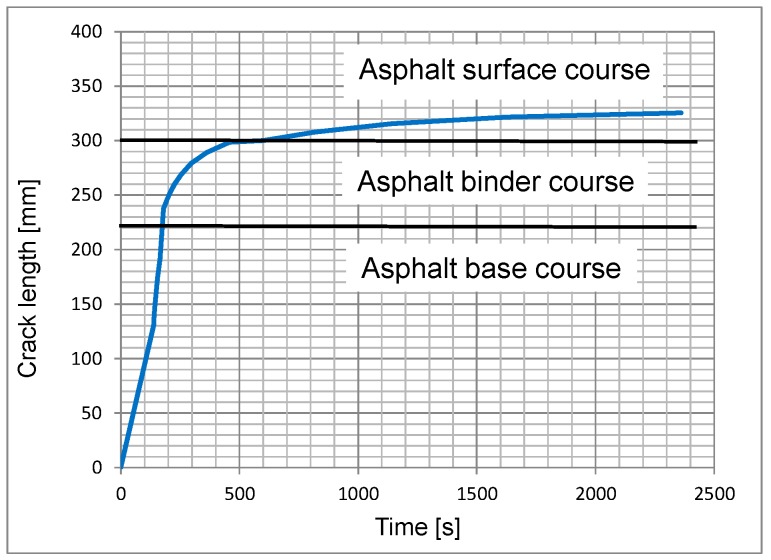
Development of the crack length as a function of time.

Figure 11 shows the crack propagation as a function of the load from the notch at the bottom of the base layer through the entire asphalt pavement. Under a steadily increasing load, the crack propagation decreases as it moves towards the surface. Initially, in the asphalt base layer, the crack propagates faster and then slows down in the asphalt binder layer. In the asphalt surface layer, the crack propagates at a relatively steady rate.

## 5. Prediction of Load Cycles to the Failure of the Asphalt Pavement Structure

Because the simulation of 3D 3PSCBT under cyclic (dynamic) loading requires extensive computational resources in ABAQUS, the crack propagation was evaluated with the aid of the CZM under static loading. In the following, the crack propagation in the static simulation and the dynamic laboratory 3PSCBT is linked based on the energy balance approach [35]. The findings are further used to draw conclusions about the fracture behavior of the asphalt pavement (i.e., permissible loading cycles to material failure). 

The energy balance approach is based on the assumption that the fracture energy takes on a constant value as the material fails, independent of the loading:

(5) $$G_{s}=G_{d}$$

with:

$G_{d}$ Fracture energy for dynamic 3PSCBT at failure (mJ) (in the laboratory),
$G_{s}$ Fracture energy for static 3PSCBT at failure (mJ) (in the simulation).

Table 5 shows the fracture energies for the static simulations as well as the dynamic 3PSCBT test. The results from the simulation yield lower fracture energies than the dynamic 3PSCBT test in every case. The discrepancy may be attributed to the assumptions that the sample is homogeneous and the linear elastic properties of the continuum elements. The fracture energy determined with the CZM may not exactly correspond to the measured fracture energy. The calibration factor CF is introduced to adjust the results:

(6) $$\mathrm{CF}=\frac{G_{d}}{G_{s}}$$

The fracture energy obtained from the dynamic 3PSCBT and the number of loading cycles N_3PSCBT_ at the failure of the sample can be used to estimate the number of loading cycles to failure N_real_ in pavement structures. (see Table 5):

(7) $$N_{\mathrm{real}}=\frac{G_{\mathrm{real}}\cdot \mathrm{CF}}{G_{d}}\cdot N_{3\mathrm{PSCBT}}$$

with:

$G_{d}$ Fracture energy for dynamic 3PSCBT at failure (mJ) (in the laboratory),
$G_{\mathrm{real}}$ Fracture energy for static loading of the pavement structure (mJ) (in the simulation),
CF Calibration factor (-) (see Table 5),
$N_{3\mathrm{PSCBT}}$ Load cycles to failure for dynamic 3PSCBT (see Table 6).

From Table 6, one can see that in contrast to the samples of the 3PSCBT, the fracture energy in the real asphalt pavement, which is required up until the break of the single layer, depends on numerous factors. In addition to the material properties and the state of stress, the layer thickness plays an important role. The fracture energy for the asphalt base layer is comparable to that for the asphalt surface layer in 3PSCBT. In the simulation of the real asphalt pavement, however, the fracture energy for the asphalt base layer is four times the fracture energy of the asphalt surface layer.

## 6. Conclusions and Outlook

This study proposed an FE modeling with a cohesive zone approach, which may deliver realistic results for the fracture processes in pavement construction materials. A new approach to transfer the fracture processes from tests on single layer samples to multi-layered pavement structures is initially developed. The results will offer better insight into the complex interrelations between the emergence of macro-cracks in the asphalt pavement and crack propagation thereafter. This will allow for the establishment of important interrelations for the prediction of the remaining service life of an asphalt pavement after the initial onset of macro-cracking, which shall ultimately serve as input with regard to the assessment of asphalt pavements and innovative design strategies.

This research focused on the crack propagation at a given temperature. Therefore, it is necessary to expand the test regime for further research at different temperatures and various loading scenarios. The respective fracture mechanical parameters are to be determined and applied in the CZM. With a sufficient set of data, a precise prediction of an asphalt pavement’s remaining service life after the emergence of macro-cracks will be made possible. The developed CZM can represent a useful augmentation to conventional design methods for asphalt pavement structures. Further efforts will be focused on the consideration of visco-elastic material properties for the continuum elements. The consideration of a multi-phase (air voids, aggregate, and mastic) will yield different and significantly different stress distributions and ultimately allow for more precise results. The moving, heterogeneous load corresponding to a tire should be implemented in the future to further approximate the pavement conditions in reality. Overall, a more realistic modeling will allow for more precise predictions of loading states and will ultimately facilitate an increase in quality, the economic feasibility of conventional pavement design methods.

## Figures and Tables

**Table 1:** Gradations of the used asphalt types.

Characteristic	Asphalt Type		
	AC 22 TS	AC 16 BS	AC 8 DS
Bitumen content (M.-%)	4.1	4.5	6.2
Aggregate	Gabbro	Granodiorite	Granodiorite
>0.063 mm	7	6	11
0.063–2.0 mm	26	18	28
2.0–5.6 mm	20	16	32
5.6–8.0 mm	12	17	27
8.0–11.2 mm	10	19	2
11.2–16.0 mm	10	24	-
16.0–22.4 mm	15	-	-
Bulk density (g/cm^3^)	2.539	2.366	2.420
Density (g/cm^3^)	2.695	2.557	2.499
Void content (%)	5.8	7.5	3.2

**Table 2:** Material parameters at +5 °C.

Asphalt Type	Modulus of Elasticity E (MPa)	Poisson’s Ratio ν (-)	Maximum Cohesive Strength T° (MPa)	Fracture Energy Density G_c_ (mJ/mm^2^)	Stiffness K° for Cohesive Elements (MPa/mm)
Asphalt surface layer (AC 8 DS)	15,743	0.30	3.45	2.00	3.85
Asphalt binder layer (AC 16 BS)	14,155	0.30	2.70	1.20	3.80
Asphalt base layer (AC 22 TS)	20,676	0.30	4.25	2.25	4.20

**Table 3:** Lower and upper stresses in the dynamic 3PSCBT.

Asphalt Type	Lower Stress (MPa)	Upper Stress (MPa)
Asphalt surface layer (AC 8 DS)	0.1	2.4
Asphalt binder layer (AC 16 BS)	0.1	2.2
Asphalt base layer (AC 22 TS)	0.1	2.9

**Table 4:** Geometry and material properties of asphalt pavement according to RStO 12.

	Thickness (mm)	Young’s Modulus E (MPa)	Poisson’s Ratio ν (-)	Maximum Cohesive Strength T° (MPa)	Fracture Energy Density G_c_ (mJ/mm^2^)	Stiffness K° for The Cohesive Elements (MPa/mm)
Asphalt surface layer	40	15,743	0.30	3.45	2.00	3.85
Asphalt binder layer	80	14,155	0.30	2.70	1.20	3.80
Asphalt base layer	220	20,676	0.30	4.25	2.25	4.20
Sub-base	310	100	0.49	-	-	-
Subgrade	7000	45	0.49	-	-	-

**Table 5:** Fracture energy for the 3PSCBT.

	Fracture Energy from the Dynamic 3PSCBT $G_{d}$ (mJ)	Fracture Energy from the Numerical Simulation $G_{s}$ (mJ)	Calibration Factor CF (-)
Asphalt surface layer	8994	6862	1.31
Asphalt binder layer	5405	4350	1.24
Asphalt base layer	9207	7720	1.19

**Table 6:** Determining the permissible number of load cycles for real pavement structures.

Asphalt Layer	$G_{d}$ (mJ)	$N_{3PSCBT}$ (-)	$G_{real}$ (mJ)	$N_{real}$ (-)
Asphalt surface layer	8994	12,885	4200	7882
Asphalt binder layer	5405	4469	13,500	13,841
Asphalt base layer	9207	10,550	19,590	26,712
